# Contrasting signals of positive selection in genes involved in human skin-color variation from tests based on SNP scans and resequencing

**DOI:** 10.1186/2041-2223-2-24

**Published:** 2011-12-01

**Authors:** Johanna Maria de Gruijter, Oscar Lao, Mark Vermeulen, Yali Xue, Cara Woodwark, Christopher J Gillson, Alison J Coffey, Qasim Ayub, S Qasim Mehdi, Manfred Kayser, Chris Tyler-Smith

**Affiliations:** 1 Department of Forensic Molecular Biology, Erasmus MC University Medical Center, PO Box 2040, Rotterdam, 3000 CA, The Netherlands; 2 Netherlands Forensic Institute, Postbus 24044, The Hague, 2490 AA, The Netherlands; 3 Wellcome Trust Genome Campus, Wellcome Trust Sanger Institute, Hinxton, Cambridge, CB10 1SA, UK; 4 Sindh Institute of Urology and Transplantation (SIUT) Karachi, Civil Hospital Karachi 74200, Pakistan

## Abstract

**Background:**

Numerous genome-wide scans conducted by genotyping previously ascertained single-nucleotide polymorphisms (SNPs) have provided candidate signatures for positive selection in various regions of the human genome, including in genes involved in pigmentation traits. However, it is unclear how well the signatures discovered by such haplotype-based test statistics can be reproduced in tests based on full resequencing data. Four genes (*oculocutaneous albinism II *(*OCA2*)*, tyrosinase-related protein 1 *(*TYRP1*)*, dopachrome tautomerase *(*DCT*), and *KIT ligand *(*KITLG*)) implicated in human skin-color variation, have shown evidence for positive selection in Europeans and East Asians in previous SNP-scan data. In the current study, we resequenced 4.7 to 6.7 kb of DNA from each of these genes in Africans, Europeans, East Asians, and South Asians.

**Results:**

Applying all commonly used neutrality-test statistics for allele frequency distribution to the newly generated sequence data provided conflicting results regarding evidence for positive selection. Previous haplotype-based findings could not be clearly confirmed. Although some tests were marginally significant for some populations and genes, none of them were significant after multiple-testing correction. Combined *P *values for each gene-population pair did not improve these results. Application of Approximate Bayesian Computation Markov chain Monte Carlo based to these sequence data using a simple forward simulator revealed broad posterior distributions of the selective parameters for all four genes, providing no support for positive selection. However, when we applied this approach to published sequence data on *SLC45A2*, another human pigmentation candidate gene, we could readily confirm evidence for positive selection, as previously detected with sequence-based and some haplotype-based tests.

**Conclusions:**

Overall, our data indicate that even genes that are strong biological candidates for positive selection and show reproducible signatures of positive selection in SNP scans do not always show the same replicability of selection signals in other tests, which should be considered in future studies on detecting positive selection in genetic data.

## Background

Large-scale genotyping projects using genome-wide single-nucleotide polymorphisms (SNPs) have provided large amounts of data describing the genetic diversity of human populations [1-6]. Several statistical methods have been developed and used for detection of signatures of selective processes from genome-wide SNP data, which we refer to as 'SNP scans' [7]. All these approaches try to recover fingerprints of selective sweeps by detecting signals in the haplotypic variation of a genomic region and/or the spectrum of the variation of the genetic diversity [8-15]. However, the results obtained with the different test statistics usually show limited overlap (see results from Voight *et al*. [12] Wang *et al*. [14] and Akey [16]), therefore, it would be desirable to compare the results from SNP haplotype-based tests using the 'gold standard' of full resequencing data and suitable statistical tests. The most widely used approach involves computing a set of neutrality-test values for sequence data from a particular genomic region, and then estimating the likelihood of such values are under neutrality. This is achieved either by comparing the computed one in the region of interest with the value of the statistic in other regions of the genome (that is, by using empirical distributions [17]) or with the values obtained under demographic (neutral) simulations [18]). However, it is also desirable to have an estimate of the selective parameters (s and h) rather than just rejecting the hypothesis of neutrality. The latter could be in principle obtained by applying Approximate Bayesian Computation (ABC) [19], a statistical technique used to recover the posterior distribution of parameters shaping the statistical model, which is applied when computing the likelihood of the data given the parameters is not possible, but it is possible to simulate data under the model of interest [19]. ABC has proven to be a valuable statistical tool for making inferences about demographic parameters in population genetics. Moreover, it has also been used in estimating selective parameters [20,21].

Skin pigmentation is an excellent candidate system for investigating positive selection. It is very likely that this trait is under selective pressure, given the biological role of pigmentation and the large differences seen in pigmentation intensity between continental populations [22]. For a large number of genes involved in the pigmentation pathway, signatures of recent selective sweeps have been suggested from SNP-scan data [12,14,23-26], and DNA sequence-based evidence has been tested for a limited number of the genes associated with human skin pigmentation (*melanocortin 1 receptor (MC1R), solute carrier family 45 member 2 (SLC45A2)*, *tyrosinase-related protein 1 (TYRP1, dopachrome tautomerase (DCT) *and *tyrosinase *(*TYR) *[27-30]. In a previous SNP-based study, we identified signatures of selective sweeps in the genes *oculocutaneous albinism II (OCA2),TYRP1, DCT, and KIT ligand (KITLG) *in Europeans, in *OCA2, DCT, KITLG, epidermal growth factor receptor (EGFR*) and *dopamine receptor D2 (DRD2) *in East Asians. In contrast, Africans did not show any evidence of positive selection in any of the genes that we tested [24]. Evidence for selection in *OCA2, DCT, KITLG*, and *TYRP1 *was also shown by other SNP-based studies applying similar haplotype-based tests to data from other samples [23-26].

In the present study, we generated DNA sequence data from approximately 4.7 to 6.7 kb of each of the four genes *OCA2, TYRP1, DCT *and *KITLG *in Africans, Europeans, East Asians and South Asians, and applied both neutrality tests and an ABC-Markov chain Monte Carlo (MCMC) approach for detecting evidence of positive selection. Furthermore, we compared the outcomes from such sequence-based test with our previous results from haplotype-based tests using SNP-scan data.

## Results

We sequenced DNA regions of approximately 4.7 to 6.7 kb from each four genes, *OCA2*, *DCT*, *TYRP1 *and *KITLG*, involved mainly in human skin pigmentation, in 24 to 26 DNA samples from African, European, East Asian and South Asian populations (Table 1). In total, we detected 146 polymorphic positions: 47 in the *OCA2 *fragment, 35 in the *TYRP1 *fragment, 31 in the *DCT *fragment and 33 in the *KITLG *fragment (Table 1).

**Table 1:** Diversity statistics estimated for four major pigmentation genes and four worldwide populations

Gene (number of base pairs sequenced)	Population	Sample size, n	Haplotypes, n	Polymorphic sites, n	Nucleotide diversity (×10^-4^)	Watterson's estimator (${\overset{\wedge }{\theta }}_{w}$)	
						Absolute number	×10^-4 ^per site
*OCA2 *(6729)	Worldwide	98	27	47			
	African (YRI)	24	14	34	12.7	7.66	11.4
	European (CEU)	24	6	23	10.9	5.18	7.7
	East Asian (CHB)	24	6	22	8.4	4.96	7.4
	South Asian (BRU)	26	10	23	10.6	5.09	7.6
*TYRP1 *(4780)	Worldwide	98	34	35			
	African (YRI)	24	15	22	7.2	4.96	10.4
	European (CUE)	24	9	15	8.1	3.38	7.1
	East Asian (CHB)	24	14	18	4.7	4.06	8.5
	South Asian (BRU)	26	7	14	8.5	3.1	6.5
*DCT *(4905)	Worldwide	97	23	31			
	African (YRI)	24	12	26	8.9	5.9	11.9
	European (CEU)	24	7	16	6.9	3.6	7.4
	East Asian (CHB)	24	7	15	9	3.4	6.9
	South Asian (BRU)	25	11	18	11.5	4	8.2
*KITLG *(5869)	Worldwide	97	26	33			
	African (YRI)	24	15	27	10.8	6.1	10.4
	European (CEU)	24	6	18	9	4.1	6.9
	East Asian (CHB)	24	8	21	10.8	4.7	8.1
	South Asian (BRU)	25	10	18	9.3	4	6.8

BRU, Brahui from Pakistan; CHB, Han Chinese from Beijing; CEU, Council for Education on Public Health Utah; YRI, Yoruba from Ibadan, Nigeria.

### Neutrality tests and their statistical significance

The application of the four-gamete rule between all pairs of SNPs within each population suggested that, for all genes except *TYRP1*, the sequenced region could be considered as a single block (see Additional file 1). We compiled results from commonly used neutrality tests under different demographic models and using an empirical distribution (Table 2), and computed the two-tailed *P *value for each statistic given the population and gene (see Additional file 2). We found discrepancies in rejecting the null hypothesis of neutrality depending on which expected neutral distribution was used. After Bonferroni correction for multiple testing (*P*<0.05/96 computed tests for each expected distribution considered under neutrality in the case of the cosi model (CM) and the Gutenkunst model (GM, a best-fit demographic model); and *P*<0.05/60 computed tests in the case of Encyclopedia of DNA Elements (ENCODE) data (ED)), Fu's Fs statistic was significant for the *TYRP1 *gene and the Han Chinese from Beijing (CHB) group when using the empirical distribution from the ED, and marginally significant (*P *= 0.045) for the *OCA2 *gene and the Council for Education on Public Health (CEPH) Utah (CEU) group when using the empirical distribution from CM. None of the neutrality tests reached statistical significance after Bonferroni correction using the CM, and only the Fu and Li D and D* statistics were significant for *KITLG *in Europeans using the GM. Combining the *P *values of the different statistics produced a different picture. We detected significant (combined *P*< 0.05) departures from neutrality in the African and European populations for *OCA2*, the African population for *DCT*, and the European population for *KITLG *using the CM, and for *KITLG *in the European population using the GM. By contrast, ED-based *P *values were significant for *TYRP1 *in the Asian populations.

**Table 2:** Neutrality-test statistics of each gene in each population.^a^

Gene	Population	Tajima's D	Fu and Li's				Fay and Wu's H	Fu's Fs	EWHT^b^	GM^c^	CM^d^	ED^e^
									
			D*	F*	D	F						
*OCA2*	African (YRI)	0.368	0.67	0.67	0.71	0.71	3.16	1.73	0.18	0.08	0.01	0.95
	European (CEU)	1.36	0.75	1.14	0.79	1.21	-1.94	9.23	0.30	0.16	0.05	0.86
	East Asian (CHB)	0.46	0.70	0.73	0.74	0.77	-5.74	6.78	0.47	0.29	0.38	0.48
*TYRP1*	African (YRI)	-0.99	0.02	-0.39	-0.02	-0.45	-7.76	-3.4	0.10	0.66	0.65	0.08
	European (CEU)	0.45	-0.70	-0.37	0.19	0.34	-5.24	1.30	0.31	0.96	0.89	0.49
	East Asian (CHB)	-1.41	-0.74	-1.15	-0.85	-1.26	-4.91	-5.20	0.23	0.33	0.19	<0.00005
*DCT*	African (YRI)	-0.83	1.18	0.57	1.28	0.60	-3.54	-0.14	0.18	0.15	<0.005	0.84
	European (CEU)	-0.20	1.16	0.83	1.23	0.87	-3.89	2.30	0.24	0.39	0.16	0.78
	East Asian (CHB)	0.95	1.12	1.25	1.18	1.32	-4.61	3.72	0.54	0.24	0.44	0.74
*KITLG*	African (YRI)	0.14	0.35	0.33	0.35	0.33	-4.35	-0.28	0.11	0.13	0.17	0.79
	European (CEU)	0.97	1.25	1.36	1.33	1.44	-4.39	6.24	0.39	4.17E-05	<0.01	0.95
	East Asian (CHB)	1.10	0.29	0.68	0.28	0.70	-2.81	5.01	0.32	0.38	0.69	0.69

^a^Neutrality-test statistics and combined *P *values for each population and gene for which a neutrality test distribution could be computed. Combined *P *values using the approach proposed by [64] were computed using three different neutrality distributions.

^b^Ewens-Watterson homozygosity test

^c^Maximum likelihood model of Gutenkunst *et al *[44],

^d^Cosi best fit to the best-fit parameters of Schaffner *et al*. [45].

^e^Encyclopedia of DNA Elements (ENCODE) data from resampled sequences from three different ENCODE regions.

### Phylogenetic networks

**Figure 1:**
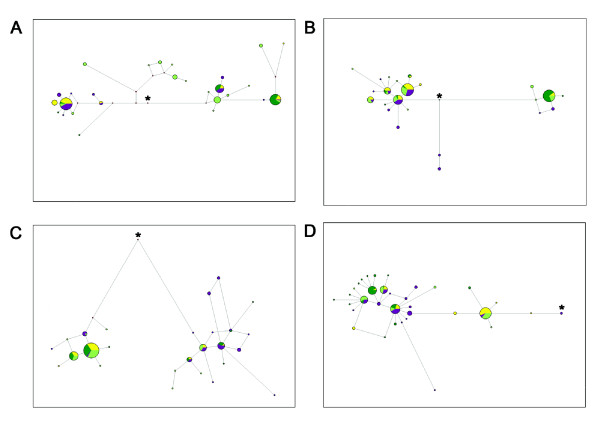
**Maximum parsimony networks (using the median-joining algorithm) inferred from sequence data of the genes (A) *OCA2*, (B) *DCT *(C), *KITLG *and (D) *TYRP1***. Each circle represents a haplotype, and has an area proportional to the haplotype frequency in European (yellow), East Asian (dark green), African (purple) and South Asian (light green) populations. Branch lengths represent the number of mutations separating the haplotypes, with the shortest branches indicating one mutation. An asterisk denotes where the ancestral haplotype joins the network as derived from sequence data of chimpanzee, gorilla, and orangutan.

To visualize the phylogenetic relationships between the haplotypes and to provide additional insights into their evolutionary history, we constructed median-joining networks. Overall, the networks had few reticulations (Figure 1). On average, the African population tended to show a large number of haplotypes at low frequency, whereas the European, East Asian and Pakistanis populations all had two main haplotypes separated by a large number of mutations. This was particularly dramatic in the case of *KITLG *and *DCT*, and less striking for *OCA2 *and *TYRP1*.

### Approximate Bayesian computation/Markov chain Monte Carlo analyses

**Figure 2:**
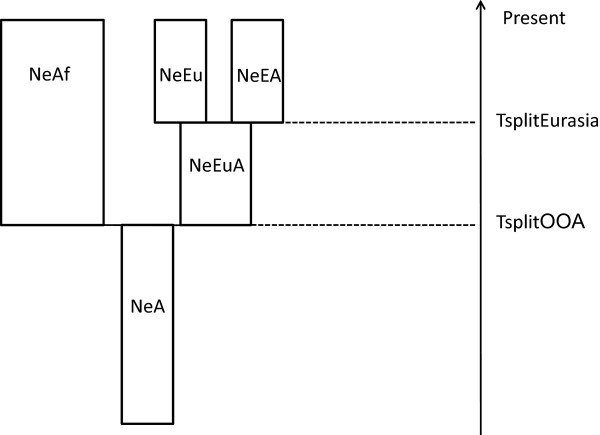
**Out-of-Africa (OOA) model implemented in the forward simulator and further used for approximate Bayesian computation (ABC) estimation**. An ancestral population with size N_eA _splits at T_splitOOA _into two new populations: Africa (with N_eAf_) and Eurasia (with Ne_EuA_), and this population at T_splitEurasia _splits in two populations, Europe (with N_eEu_) and Asia (with N_eEA_).

We first estimated demographic parameters for our simplified OOA (out-of-Africa) model (Figure 2) considering 50 loci from the Environmental Genome Project; the statistics of centrality and dispersion of the different parameters are described (Table 3). In all cases, the posterior distributions strongly diverged from the priors (which were all uniform) (Table 3), and thus were influenced by the data. Posterior estimates of the parameters related to a putative selective event for each of the four genes under study are shown (Table 4). As controls, we also performed ABC-MCMC with data from *SLC45A2 *and from a simulated neutral region of 5 kb. Histograms of the posterior distributions for each gene are provided (see Additional file 3). Posterior distributions of the parameters were similar to the prior distributions in all the cases except for *SLC45A2*; in this case, selection was restricted to Europeans, beginning after the split from East Asians and fitting a dominant model of inheritance (*h *= 1). The estimated posterior distribution of σ was skewed towards large values.

**Table 3:** Median and dispersion statistics of the posterior distributions of the demographic parameters

Parameter	Prior	Median	95% CI
Ne_YRI_	U_(500,10000)_	5070	(2400 - 8930)
Ne_CEU_	U_(500,5000)_	1630	(130 - 6900)
Ne_CHB_	U_(500,5000)_	4720	(1920 - 7650)
Ne_CEU_CHB_	U_(500,5000)_	4390	(1420 - 8890)
Ne_A_	U_(500,5000)_	4800	(2160 - 8900)
t_SplitCEU_CHB_	U_(0.17, 0.83)_^b^	45,250	(11,500 - 63,250)
t_SplitOAA_	U_(100,000, 325,000)_	52,250	(50,250 - 76,000)

CI, confidence interval.

^a^Estimated by approximate bayesian computation/Markov chain Monte Carlo (ABC-MCMC) from regions of 10 kb each from 50 genes from the Environmental Genome Project database with a burn-in of 1,000 simulations, 9,000 retained simulations and a thinning of 9. The total number of iterations retained for estimating the posterior distributions was 1,000. Times are computed in years as the number of generations multiplied by an estimated generation time of 25 years, and a scaling factor of 10 that was used (μ was set constant to 2.35 × 10^-7 ^after scaling by 10). Effective population sizes are in number of diploid individuals after correcting by the scaling factor of 10.

^b^Time of split of CEU and CHB as a function of the remaining time after the out-of-Africa split. A value of 1 would indicate that CEU and CHB split just after the out-of-Africa split.

**Table 4:** Median and 95% Credible Interval (CI) of the estimates of the time when selection started

Gene	Population under positive selection^a ^	Time of selection (95% CI)	σ (95% CI)	*h *(95% CI)
*TYRP1 *	Europe^b^	50,250 (13,500 to 97,250)	361.94 (55.6 to 984.79)	1.70 (0.71 to 4.28)

*OCA2 *	Europe,^b ^East Asia	45,875 (12,750 to 95,006)	383.42 (36.07 to 992.04)	2.04 (0.78 to 4.56)

*KITLG *	Europe,^b ^East Asia^b^	46,000 (11,750 to 93,269)	385.49 (37.68 to 1036.32)	2.01 (0.74 to 4.41)

*DCT *	East Asia^b^	48,500 (11,250 to 97,006)	789.24 (55.21 to 1584.98)	1.90 (0.67 to 4.11)

*SLC45A2 *	Europe^b^	27,500 (10,500 to 73,294)	676.53 (127.73 to 1026.06)	1.10 (0.53 to 2.60)

Neutral simulation	Europe^b^	44,875 (12500 to 95,756.25)	593.18 (47.99 to 1086.22)	1.69 (0.56 to 3.97)

^a^The table shows the selection parameter σ (defined as 4 × N_e _× s, where N_e _is the harmonic mean of the effective population size through time) and overdominance parameter *h *computed for each population that has been suggested as being under selective pressures in the genes that have been sequenced, data from Soejima *et al*. [30] for *SLC45A2*, known to be under selective pressure in Europeans (see Methods), and 5 kb produced under a neutral out-of-Africa model (s = 0, *h *= 0).

^a^From Lao *et al*. 2007. Prior distributions (without scaling) were truncated normal (mean ± sd 1810 ± 800 generations, range 400 to 4000) for the time of selection, truncated normal (mean ± sd -4 ± 3, range -4 to 2) for s in logit form, and truncated normal (mean ± sd 1 ± 1, range 0.5 to 6) for *h*. In total, 2,000 simulations were performed for each gene using as a prior distribution of the demographic parameters the median value of the posterior distributions obtained from the 50 loci (see Methods). A burn-in of 1,000 simulations was used.

^b^Population where the selective pressure was simulated.

## Discussion

In the present study, we focused on reinvestigating previous conclusions about positive selection based on long-range haplotype (LRH) tests, using four genes putatively associated with human pigmentation. The phylogenetic networks for each gene based on sequence data were in agreement with our previous findings [24]; the different populations tended to show a high frequency of one of the major haplotypes, which tended to diverge from the others by a large number of mutations, and the single SNP differentiation between populations was also in agreement with previous results. This was particularly evident for *OCA2*, *KITLG *and *DCT *in European and Asian populations, and less evident in the African population. The Pakistani population, geographically situated between the Europeans and Asians, shares the main haplotypes with these two populations. The presence of long network branches within each population can be indicative of balancing selection [31]; however, we failed to replicate previous LRH findings with the sequence-based tests, and we observed dependence of the statistical significance of the sequence-based tests on which neutrality distribution was used. Only the *KITLG *in the European population had statistical departures from neutrality in the CM and GM, which is in agreement with the outcome from the LRH test, but neutrality could not be rejected using ENCODE data. Furthermore, we were not able to replicate a previously suggested signal for the *DCT *gene in Asian populations [29]. As the whole *DCT *gene is a single linkage disequilibrium (LD) block, as seen in the HapMap East Asian data, it seems unlikely that the discrepancy is explicable by the different *DCT *regions sequenced. Indeed, the agreement between different SNP-scan studies has been described as 'underwhelming' [16].

The discrepancies we detected here between haplotype-based and sequence-based test outcomes can be explained by a number of factors.

First, we cannot exclude the possibility that the positive-selection signals from our previous SNP-based study were false positives; the complex demographic history of humans [12,32,33] and the power dependency of the tested site [34] can affect the outcome of such tests.

Second, it has been emphasized that the SNP ascertainment bias introduced during marker discovery [35,36] and genotyping array can lead to spurious false-positive findings in haplotype-based tests [37,38].

Third, there might be a lack of power in the sequence-based tests because of the small sample sizes and/or small sequenced regions [39]. Although we cannot exclude this possibility, the length (approximately 5 kb) sequenced from the four genes proved to be sufficient to detect departures from neutrality in *SLC45A2 *in the European population (data not shown).

A fourth possibility is that the distributions that we computed for each statistic under neutrality do not represent the true underlying distribution for the human species. Parameters of the demographic events need to be defined *a priori*, which in humans is challenging because of the complex history of migrations, admixture, expansions and bottlenecks [40]. The differences seen in the values of the parameters could be indeed a major source of variation. The ENCODE data we used as an alternative is hampered by the fact that the considered regions were ascertained based on their genomic peculiarities [41], and they may not be representative of the genetic variability of the genome. There has been progress in resequencing entire genomes (for example, the 1000 Genomes Project; http://www.1000genomes.org/page.php); however, current projects rely on combining low-coverage data from multiple samples, and are not able to produce the accurate sequence for each genome that is needed for such comparisons [42].

The fifth, and perhaps most likely, reason for discrepancies between LRH and sequence-based tests we observed here may be the different underlying assumptions of the evolutionary models used (that is, instantaneous selective sweep versus incomplete selective sweeps) in the definition of each statistic, and the evolutionary timescale over which each type of test can recover departures from neutrality [7]. In that case, our results might indicate an extremely recent selection in the pigmentation genes, which would be recovered by haplotype-based but not sequence-based tests.

We also used a Bayesian approach to estimate selective parameters of the populations putatively under positive selection in each gene. The demographic parameters were on average in concordance with those described in previous studies [43-46]; however, it should be noted that they were not entirely comparable as there were a large number of differences in the assumptions of the models and data. Despite this technical limitation of the approach, the estimates of the time when selection started and the mode of inheritance correlated well with expectations in the case of *SLC45A2 *[30], independently of whether the complete 10 kb sequence or a subsample of 5 kb was used (data not shown). To our knowledge, this is the first time this known selective sweep has been quantified in such a way. However, for *OCA2*, *TYRP1, DCT *and *KITLG *and the neutral simulated region, the strong resemblance between the prior and the posterior distributions suggests that the latter are mainly dominated by the priors rather than by the information contained in the genetic data.

## Conclusions

In this study, we have shown that using sequence-based neutrality tests to confirm signatures of positive selection derived from LRH tests on SNP-scan data can be difficult, even though there is a strong likelihood that the skin-pigmentation genes we studied have been targets of selection [47]. Deciphering whether this is a consequence of the power of the different statistics to detect the fingerprint of selection on different timescales; different assumptions on the strength of the selective event; or lack of power due to experimental limitations seems challenging.

Our findings should be considered in future studies that set out to further investigate signatures of selection in other genes or regions of the human genome suggested by SNP-scan data. It may be argued that the final proof of positive selection should not be provided by additional genetic data but rather by functional evidence, but this may have its own caveats. A recent study [48] has demonstrated that the *TRPV6 *gene shows strong evidence of positive selection in all non-African populations tested using a novel modification of the extended haplotype homozygosity (EHH) test, but no functional differences between the ancestral and derived sequence could be detected using experiments relevant to the known gene function. Clearly, further advancements in the methods used to detect and validate putative signatures of positive selection are needed, and provide one of the most exciting areas for future developments. Complete understanding of positive selection in the human genome will require the combination of multiple lines of evidence.

## Methods

### Population samples

Genomic DNAs (Coriell Institute for Medical Research; Camden, NJ, USA from randomly ascertained participants from three continents chosen from the HapMap panel [1] were used: 24 Yoruba from Ibadan, Nigeria (YRI); 24 Han Chinese from Beijing (CHB); and 24 CEPH Utah residents with ancestry from northern and western Europe (CEU). In addition, 26 (*OCA2 *and *TYRP1*) or 25 (*DCT *and *KITLG*) Brahui (BRU) from Pakistan [49] were included because of their distinct and intermediate geographic location and history. In some analyses, we used published data on *SLC42A2 *(Table 3 of Soejima *et al*.[30]) in European-Africans from Cape Town, Ghanaians from Accra, and Chinese from Guangzhou; these data had excluded SNPs at a frequency of <5%. Genomic DNA samples from one chimpanzee (*Pan troglodytes*), one orangutan (*Pongo pygmaeus*) and one gorilla (*Gorilla gorilla*) (all from the European Collection of Cell Cultures, Salisbury, Wiltshire, UK) were also included to allow inference of the ancestral state of each SNP.

### Ascertainment of the sequenced regions

**Figure 3:**
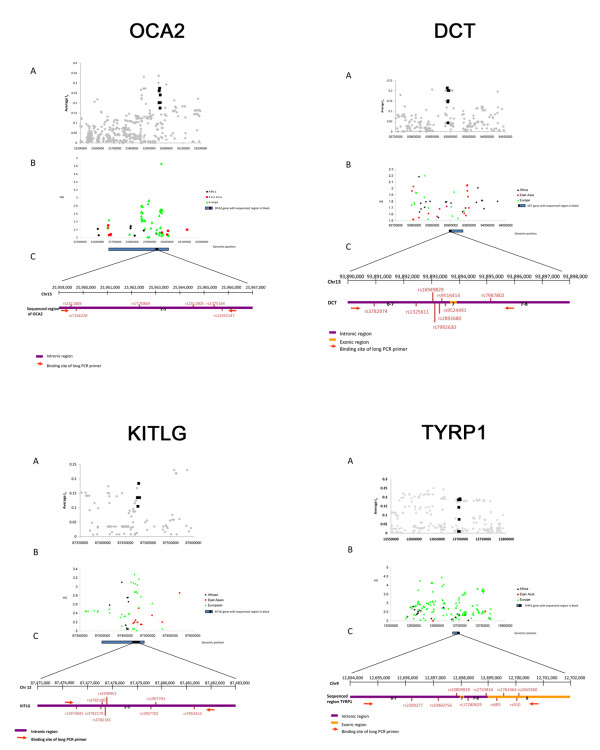
**Regions of the *OCA2*, *DCT*, *KITLG *and *TYRP1 *genes analyzed**. (A) Mean amount of informativeness of a window centered on each SNP [24] for the three PERLEGEN populations. Black dots indicate SNPs present in PERLEGEN from the sequenced region. **(B) **Integrated haplotype score (iHS) statistic obtained from Happlotter [12] in the three HapMap populations for the region of interest. **(C) **Representation of the sequenced region.

For each of the four genes included in this study (*OCA2*, *DCT*, *TYRP1 *and *KITLG*) a DNA region of approximately 4.7 to 6.7 kb surrounding the most informative SNP was selected for resequencing (Table 1). The region of interest was chosen based on its high rate of differentiation (quantified by means of the mean informativeness of ancestry [50] in the region) between East Asians, Europeans and Africans (Figure 3). As established previously [24], any candidate for explaining the differences between the amounts of pigmentation should genetically co-vary with such differences. Further EHH analyses suggested that these regions showed evidence of selective sweeps [24]. Additional evidence linking skin-pigmentation phenotype with the ascertained region is available for *TYRP1*[51]; an LD *r*^2 ^value of 0.704 was seen between *rs2733832*, which is associated with pigmentation, and *rs683*, which lies within the region ascertained for *TYRP1 *in HapMapII CEU. The SNPs included in the ascertained regions were rs2311806, rs7166228, rs7170869, rs2311805, rs1375164 and rs12442147 for *OCA2*; rs2209277, rs10960756, rs10809828, rs17280629, rs2733834, rs683, rs2762464, rs910 and rs1063380 for *TYRP1*; rs3782974, rs1325611, rs16949829, rs7992630, rs9516414, rs9524491, rs2892680 and rs7987802 for *DCT;*and rs1873681, rs3782179, rs3782180, rs3782181, rs4590952, rs1907702, rs1907703 and rs7953414 for *KITLG*. Although most of the ascertained regions consisted of intronic sequence, the *TYRP1 *and *DCT *fragments also contained exonic sequence: *OCA2 *(introns 2 to 3), *TYRP1 *(introns 6 to 7 and 7 to 8 plus exons 7 and 8), *DCT *(introns 6 to 7 + exon 7 + introns 7 to 8) and *KITLG *(introns 2 to 3).

### Enzymatic amplification by PCR

Primers were designed from the human reference sequence obtained from GenBank (*OCA2* accession number NC_000015.8; *TYRP1* accession number NC_000009.10; *DCT* accession number NC_000013.9 and *KITLG* accession number NC_000012.9), and used to amplify fragments of approximately 6 to 7 kb (positions on chromosome: *OCA2* 25959080-25966266; *TYRP1* 12694505-12700830; *DCT* 93890084-93895889; *KITLG* 87482044-87476046) covering the chosen regions. PCR assays were performed using a volume of 25 μl containing approximately 40 ng of genomic DNA, 2 μmol/l of each primer, 200 μmol/l of each dNTP, 2 mmol/l MgSO_4_, 0.5 U high-fidelity Taq polymerase (Platinum Taq; Invitrogen Corp., Carlsbad, CA, USA). Amplification was performed in a themal cycler (Peltier; MJ Research Inc., Waltham, MA, USA) using the following cycling profile: 94°C for 2 minutes, followed by 35 cycles at 94°C for 30 seconds, 59°C (*OCA2*, *DCT* and *KITLG*) or 56°C (*TYRP1*) for 1 minute, and 72°C for 1 minute, and a final step at 72°C for 5 minutes. Subsequently, 3 μl samples of a 1:200 dilution of these PCR products were used as templates to reamplify overlapping fragments with sizes of approximately 350 to 700 bp. PCR assays for reamplification were performed in a volume of 15 μl containing 2 μmol/l of each primer, 1.6 mmol/l MgCl_2_, 200 μmol/l of each dNTP, and 0.5 U Taq (Platinum Taq; Invitrogen). The cycling conditions for the reamplification were 94°C for 2 minutes, followed by 31 cycles at 94°C for 45 seconds, 60°C for 45 seconds, and 72°C for 1.5 minutes; then 72°C for 3 minutes. Details of all primers used in this study are given in Additional File 4.

### DNA sequencing

PCR products were purified (ExoSAP-IT; GE Healthcare, Princeton, NJ, USA) before sequencing. The primers used for reamplification (see Additional File 4) were used (individually) to sequence in both orientations, using a large-scale sequencing facility (Wellcome Trust Sanger Institute) with standard capillary methods. For each individual, each nucleotide position was determined from both strands by at least two reads each. The genomic DNA sequence for *OCA2 *(accession number NC_000015.8), *TYRP1 *(accession number NC_000009.10), *DCT *(accession number NC_000013.9) and *KITLG *(accession number NC_000012.9) were obtained from GenBank and used as the reference sequence for the relevant gene. Sequence traces were processed using the software program ExoTrace (Wellcome Trust Sanger Institute; http://www.sanger.ac.uk/ humgen/exoseq/analysis.shtml). Potentially polymorphic positions were flagged by the program, and were then checked manually. Variable positions were compared in overlapping and complementary reads for all samples. A quality-control test was performed to verify the SNPs called in each gene by comparing the genotype assignment performed by one investigator with the same genotype assignment performed by a second investigator. This test was performed on 2 kb of sequence per gene. The largest discrepancies were seen in the gene *TYRP1*, (3.4% discrepancies over all the polymorphic sites examined) and *DCT *(1.3%); *KITLG *and *OCA2 *rates of 0.28% and 0.6%, respectively. All of these discrepancies reflected differences in investigator interpretation, and all could be resolved by re-examining the traces. Therefore, for the rest of the sequenced regions, a maximum number of plausible variants was first identified by the two investigators, and the genotypes were then confirmed by a third investigator. In addition, genotypes of previously identified SNPs were compared with the genotype of the same individual in the HapMap database; 97% of the genotypes corresponded with each other, which is similar to the accuracy of the HapMap data themselves [1].

### Data analysis and coalescent simulations

Haplotypes were reconstructed using PHASE software (version 2.02 (http://depts.washington.edu/uwc4c/express-licenses/assets/phase/) [52,53]. The four-gamete rule was computed for each pair of loci in each gene. A value of 1 was assigned when the four-gamete rule could not be rejected and 0 when it could. LDheatmap http://stat-db.stat.sfu.ca:8080/statgen/research/LDheatmap/[54] was used to plot the matrix of loci for each gene. Measures of genetic diversity, including nucleotide diversity (π) [55] and Watterson's estimator, ${\overset{\wedge }{\theta }}_{w}$[56], were calculated with DnaSP (version 4.10; http://www.ub.edu/dnasp) [57]. Maximum parsimony networks (using the median-joining algorithm) were constructed using the Network 4.1 software package http://www.fluxus-technology.com. Tajima's D [58]; Fu and Li's D* and F* (no outgroup), and D and F (with outgroup [59]; Fay and Wu's H [60]; Fu's Fs [61]; and Ewens and Watterson homozygosity-test [62] statistics were estimated using a custom script to automate the computations. To test the reliability of these computations, we first compared the results obtained using our script with those obtained with DnaSP (version 4.10) [57] for some dummy data files, and saw no discrepancies. The observed values of these neutrality-test statistics in the European, Asian and African populations were compared with the values of the same neutrality-test statistics based on 10,000 coalescent simulations under the best-fit complex demographic history assuming an OOA model (the CM) implemented in the cosi software package (http://www.broadinstitute.org/~sfs/cosi/) [45]. We also estimated the departure of neutrality using the demographic model proposed by Gutenkunst *et al*. (GM) using the ms software with the syntax described in that paper ([44], supplementary material). For each statistic, a two-tailed *P *value was calculated [63] and for each population and gene, a combined *P *value was obtained [64].

### Data analysis by comparison with ENCODE data

DNA sequence information from three different regions of 500 kb each, free or almost free of genes, were obtained from the ENCODE project (EP; http://www.genome.gov/10005107) [41], comprising regions *Enr112*, *Enr113 *and *Enr213*. These regions have been resequenced by the ENCODE project in several samples from the populations (YRI, CEU, CHB, and JPT) present in the HapMap project [1]. We then performed 10,000 re-samples for each population (YRI, CEU, and CHB) and gene by dividing each of the 500 kb regions into bins of size corresponding to the sequenced region of interest, and taking at random the same number of chromosomes per population as sequenced in each of the four genes. For each bin, we computed Tajima's D, Fu and Li's D*, Fu and Li's F*, Fu's Fs and the Ewens-Watterson homozygosity-test statistics, and compared them with those computed in the gene and population of interest. These statistics were preferred over the others because they do not require the ancestral state of each SNP, which is difficult to estimate for some of the SNPs found through the ENCODE project.

### Approximate Bayesian computation/Markov chain Monte Carlo

We also implemented an MCMC without likelihoods in a similar way to the algorithm proposed by Marjoram et al. [65]. For details of the implementation of the ABC-MCMC, choice of summary statistics and the forward simulator we have used, see Additional File 1.

To estimate the strength of s and *h *and the time when selection started in each of the four genes putatively under selection, the *SLC45A2 *gene (both the full sequenced region and a randomly ascertained region of 5 kb), and the outcome from a neutral simulation of 5 kb with the OOA model (s = 0, *h *= 0), we used a two-step approach. In the first step, we performed *ABC-MCMC *using observed data for 50 regions of 10 kb each from the genes sequenced (see Additional file 5) in the same three populations by the Environmental Genome Project [66], which has previously been used to quantify demographic parameters [44]. Absence of recombination in each 10 kb segment was tested by applying the four-gamete rule in each population. The demographic model we used here is a simplification of the OOA model (Figure 2) [67]. The choice of a simple model in our study was motivated by the computational time of the forward simulations when increasing the complexity of the demographic parameters (such as bottlenecks and population expansions). As the topology of the coalescence tree is defined with a small number of sequences [68], but estimating the number of differences between pairs of sequences is performed in quadratic time, we only used 10 samples per population in order to minimize the number of computations. All the demographic parameters were scaled by a factor of 10 to reduce computational time [69] and the mutation rate per nucleotide was thus 2.35 × 10-^7^. In total, 10,000 simulations were performed, with a burn-in of 1,000 and a thinning of 9. Prior distributions for all the demographic parameters were uniform (Table 3). In the second step, we set as the constant the demographic parameters with the median value from the estimated posterior distributions, and estimated the selective parameters of each of the genomic regions under study, using all the chromosomes per population. For each genomic region, the population under selection was set as the population previously described as being under selective pressure. In all the analyses, prior distributions (without scaling) were: truncated normal (mean ± sd 1810 ± 800 generations, range 400 to 4000) for t_sel_, truncated normal (mean ± sd -4 ± 3, range -4 to 2) for *s *in logit form, and truncated normal (mean ± sd 1 ± 1, range 0.5 to 6) for the *h *parameter. In total, 20,000 simulations were performed with a burn-in of 5,000 and a thinning of 15. Prior distributions of *s*, *h *and t_sel _under selection were obtained by taking a sample from the prior distributions, performing a simulation, and retaining the sampled values if the simulated data contained the mutation under selection. Using this procedure, 1,000 simulations were retained. We repeated the procedure twice, considering CEU and CHB as being under positive or balancing selective pressures depending on the overdominance parameter.

## List of abbreviations

ABC: approximate Bayesian computation; BRU: Brahui from Pakistan; CEPH: Council for Education on Public Health; CEU: CEPH Utah; CHB: Han Chinese from Beijing; CI: Credible Interval; ED: Encyclopedia of DNA Elements (ENCODE) data; EHH: extended haplotype homozygosity; JPT: Japanese from Tokyo; LD: linkage disequilibrium; LRH: long-range haplotype test; MCMC: Markov chain Monte Carlo; OOA: out of Africa; PCR: polymerase chain reaction; SNP: single-nucleotide polymorphism; YRI: Yoruba from Ibadan, Nigeria.

## Competing interests

The authors declare that they have no competing interests.

## Authors' contributions

O. L. carried out most of the data analyses including analysis design and development and data interpretation, and took the lead in writing the manuscript. J. M. dG., with support from the Wellcome Trust Sanger Institute large-scale sequencing facility, took the lead in acquisition of data, carried out network analysis, and contributed to manuscript drafting. Y. X. was involved in acquisition of data, and provided input on the manuscript. M. V., C. W., C. J. G., A. J. C. and Q. A. were all involved in acquisition and preparation of the data. M. K. and C. T-S. designed the study, provided various resources, and were involved in data interpretation and manuscript writing. All authors have read and approved the final manuscript.

## Supplementary Material

Additional file 1 **Description of the approximate bayesian computation/Markov chain Monte Carlo (ABC-MCMC) implemented in this study**. Linkage disequilibrium (LD) plots of the considered regions. Click here for file

Additional file 2 ***P *values for each test**. Click here for file

Additional file 3 **Histograms of the posterior distributions (in black) of the selective parameters (σ (4 × N_e _× s), *h *and t_sel_) of **(A) ***SLC45A2***, (B) ***OCA2***, (C) ***DCT***, (D) ***TYRP1 ***, (E) ***KITLG *and **(F) **a neutral simulated sequence**. These should be compared with the histograms of the prior distributions (in red) for the same parameters, using as population under selective pressures **(A,B,D,E) **the Council for Education on Public Health Utah (CEU) and **(D) **East Asian populations. Click here for file

Additional file 4 **Details of all primers used in this study**. Click here for file

Additional file 5 **Environmental project genes used to fit the demographic model of the approximate bayesian computation**. Click here for file
