# Supplementary material for: Contrasting signals of positive selection in genes involved in human skin-color variation from tests based on SNP scans and resequencing
Source: Investig Genet. 2011 Dec 1;2:24. doi: 10.1186/2041-2223-2-24 (PMC3287149; doi:10.1186/2041-2223-2-24)
Supplement: Additional file 1 — Description of the approximate bayesian computation/Markov chain Monte Carlo (ABC-MCMC) implemented in this study. Linkage disequilibrium (LD) plots of the considered regions. [file 2041-2223-2-24-S1.DOC]

Supplementary material

## ABC

In the ABC MCMC algorithm described by Marjoram et al (REF), the probability
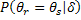
 that the proposed set of parameters (θ*_s_*) were the real parameters (*θ_r_*) was first computed. The probability function they proposed was:


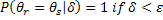


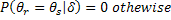


where ε was a pre-defined threshold. Then they accepted the proposed set of parameters ' with a probability h:


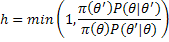


Where π(*θ*) corresponds to the prior probability of the set of parameters *θ*. Here we applied the algorithm for proposing a set of parameters given a previous one as suggested by [83], which optimizes the acceptance rate of the proposed parameters, therefore reducing the number of required iterations.

## Forward simulator

We implemented a simple forward simulator in JAVA. The main difference between this and other forward simulators [78] is that the present one does not consider recombination; this allows us to apply a forward - backward strategy to obtain simulations of the population gene genealogies by first simulating the whole genealogy of the population going forward and storing it in memory. Then, a sample of individuals is chosen from the present and the genealogy of the sequences recovered by going backward in time through the stored genealogical tree of the population. Finally, mutations are added to the genealogical tree of the sample as in coalescent backward simulators [79]. Because of this procedure, our method does not require a "burn-in" period [80] and tests whether it is possible to recover the MCRA of the sample with the number of performed generations. The basic demographic characteristics implemented in this forward simulator are: (*i*) diploidy of individuals, (*ii*) equal proportions of the two sexes, and (*iii*) random mating. For each generation, each new individual is constructed by choosing one male and one female from the previous generation according to their relative fitness, and then taking at random one chromosome from each parent. Population subdivision, migrations, expansions and contractions as well as selective pressures (both positive and negative) can be modeled in the current implementation of the program. In particular, selective events are allowed by specifying the time t_sel_ where the selective pressure starts, the selection coefficient *s*, the overdominance parameter *h* and the (sub)population where the selective event occurs. Once a selective event is established, all the further descendant (sub)population(s) also experience the selective pressure. At t_sel_, a mutation is added to one chromosome of the (sub)population(s) under selective pressure and the fitness of the individuals is then modified depending on whether or not they carry the mutation, as follows: 1 for non-carrier homozygotes, 1 + *s***h* for heterozygotes and 1+ *s* for homozygote carriers [81]. Positive *s* values indicate the presence of positive selection pressures; small positive *s* values and large *s***h* values indicate presence of strong overdominance pressures. Negative values imply purifying selection. The relative fitness of one individual (with respect to the other individuals of the same sex) is computed as his or her fitness divided by the sum of the fitness of all the individuals of the same sex. When considering a model without selective events, we applied a backward strategy, only computing the backward genealogy of the sample of individuals, but maintaining all the characteristics of the forward simulator. This reduced considerably the number of ancestors to be simulated.

## Choice of summary statistics

In order to perform ABC, a suitable summary statistic or series of summary statistics must be provided, so that the data can be compared with the simulated data. Since the outcome of a Principal Component Analysis can be interpreted in genealogical terms when the number of loci is large [87], we have used the outcome of a classical Multidimensional Scaling (MDS) computed with a matrix of mean distances between pairs of sequences as summary statistic for the analyses comprising more than one locus. In order to compare the MDS from the simulated data and the observed data, we performed a 180 degree rotation when needed, so the mean value of the first MDS variable for African sequences was positive and the mean value of the second MDS variable for European sequences was positive. After these two rotations, the variables of the MDS are still independent. The distance between these two MDS was computed for each population using a modified version of the Hausdorff distance [88] which quantifies how different are two clouds of points. For the parameters of the selective event (time of selection, selection coefficient and overdominance parameter) we computed for each population the neutrality test statistics: Tajima's D [73], Fay and Wu’s H [75], Fu and Li's D, D*, F, F* [74, 76] and Zeng's E [59]. Since the magnitude of the statistic varies, we performed the inverse of a logit transformation in order to limit all the values to the range [0,1] and avoid that the statistic with the largest magnitude dominate the final computed distance between observed and simulated summary statistics. Discrepancy between the observed statistics and the statistics from a simulation was computed as the absolute difference between them. Final distance was averaged by the number of tests that had been compared between observed data and simulated data. Distance cutoffs were estimated *ad hoc* for each simulation. In the case of the modified Hausdorff distance, we used a cutoff of 2, whereas in the case of the neutrality test statistics, we used a cutoff of 0.2.

## Four gamete rule application to each pair of SNPs in each gene

OCA2

KITLG

DCT

TYRP1
