# Supplementary figures and images for: Contrasting signals of positive selection in genes involved in human skin-color variation from tests based on SNP scans and resequencing
Source: Investig Genet. 2011 Dec 1;2:24. doi: 10.1186/2041-2223-2-24 (PMC3287149; doi:10.1186/2041-2223-2-24)

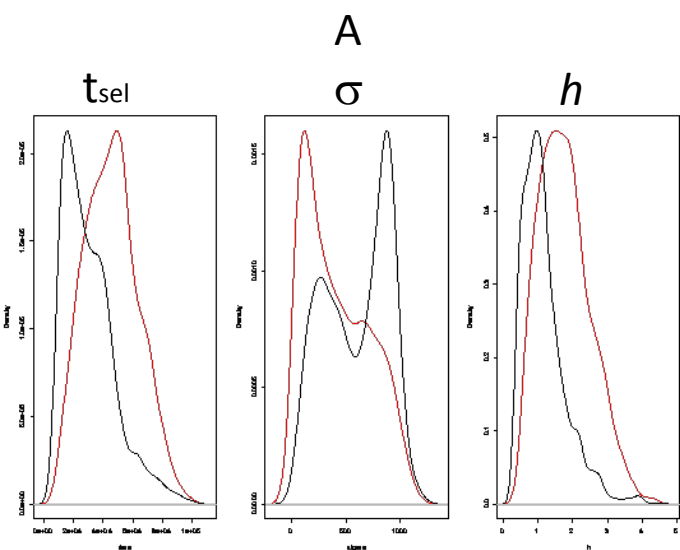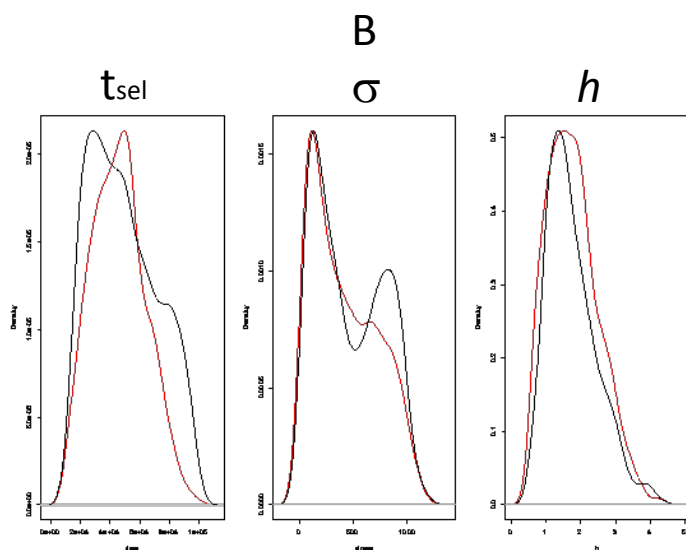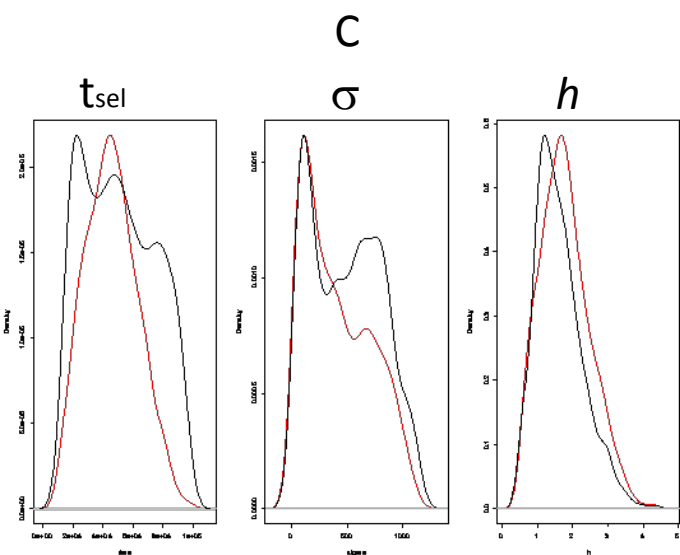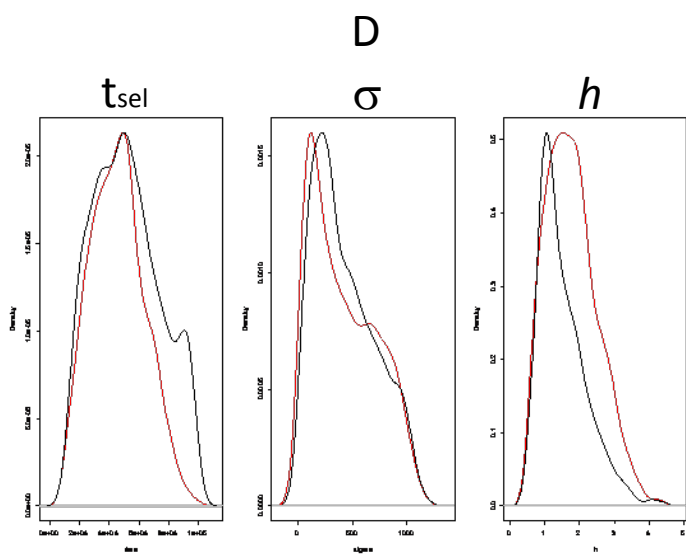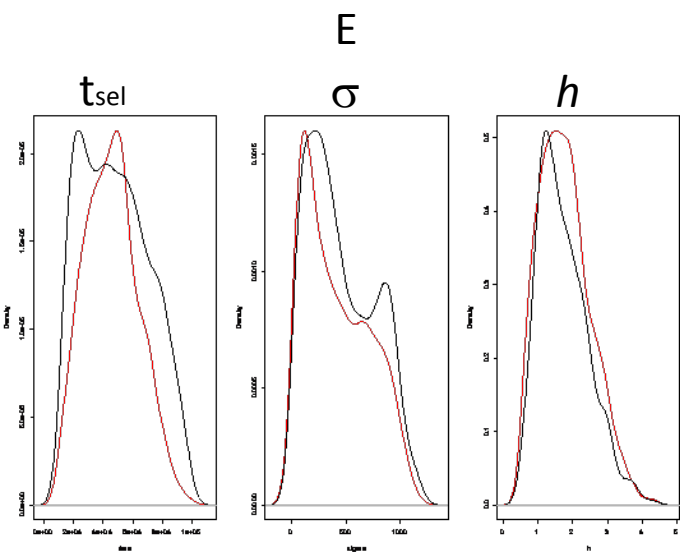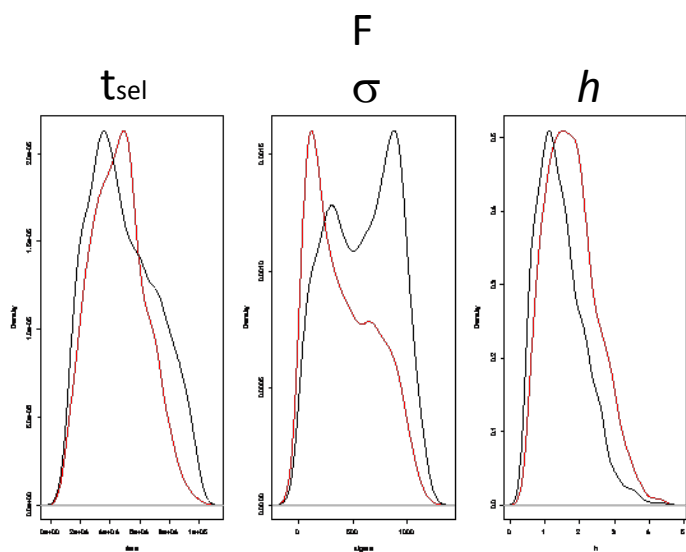

Supplement: Additional file 3 — Histograms of the posterior distributions (in black) of the selective parameters (σ (4 × Ne × s), h and tsel) of (A) SLC45A2, (B) OCA2, (C) DCT, (D) TYRP1 , (E) KITLG and (F) a neutral simulated sequence. These should be compared with the histograms of the prior distributions (in red) for the same parameters, using as population under selective pressures (A,B,D,E) the Council for Education on Public Health Utah (CEU) and (D) East Asian populations. [file 2041-2223-2-24-S3.PDF]
